# Hepatic *Acat2* overexpression promotes systemic cholesterol metabolism and adipose lipid metabolism in mice

**DOI:** 10.1007/s00125-022-05829-9

**Published:** 2022-11-15

**Authors:** Zhimin Ma, Zhengyun Huang, Chi Zhang, Xiangpeng Liu, Jie Zhang, Hui Shu, Yue Ma, Zhiwei Liu, Yu Feng, Xiyue Chen, Shihuan Kuang, Yong Zhang, Zhihao Jia

**Affiliations:** 1 Endocrinology Department, Suzhou Science & Technology Town Hospital, Suzhou, China; 2 Cambridge-Suda Genomic Resource Center, Suzhou Medical College, Soochow University, Suzhou, China; 3 Department of Endocrinology, The Second Affiliated Hospital of Soochow University, Suzhou, China; 4 Department of Animal Sciences, Purdue University, West Lafayette, IN USA; 5 Center for Cancer Research, Purdue University, West Lafayette, IN USA

**Keywords:** ACAT2, Adeno-associated virus, Bile acid, Cholesterol metabolism, Obesity

## Abstract

**Aims/hypothesis:**

Acetyl coenzyme A acetyltransferase (ACAT), also known as acetoacetyl-CoA thiolase, catalyses the formation of acetoacetyl-CoA from acetyl-CoA and forms part of the isoprenoid biosynthesis pathway. Thus, ACAT plays a central role in cholesterol metabolism in a variety of cells. Here, we aimed to assess the effect of hepatic *Acat2* overexpression on cholesterol metabolism and systemic energy metabolism.

**Methods:**

We generated liver-targeted adeno-associated virus 9 (AAV9) to achieve hepatic *Acat2* overexpression in mice. Mice were injected with AAV9 through the tail vein and subjected to morphological, physiological (body composition, indirect calorimetry, treadmill, GTT, blood biochemistry, cardiac ultrasonography and ECG), histochemical, gene expression and metabolomic analysis under normal diet or feeding with high-fat diet to investigate the role of ACAT2 in the liver.

**Results:**

Hepatic *Acat2* overexpression reduced body weight and total fat mass, elevated the metabolic rate, improved glucose tolerance and lowered the serum cholesterol level of mice. In addition, the overexpression of *Acat2* inhibited fatty acid, glucose and ketone metabolic pathways but promoted cholesterol metabolism and changed the bile acid pool and composition of the liver. Hepatic *Acat2* overexpression also decreased the size of white adipocytes and promoted lipid metabolism in white adipose tissue. Furthermore, hepatic *Acat2* overexpression protected mice from high-fat-diet-induced weight gain and metabolic defects

**Conclusions/interpretation:**

Our study identifies an essential role for ACAT2 in cholesterol metabolism and systemic energy expenditure and provides key insights into the metabolic benefits of hepatic *Acat2* overexpression. Thus, adenoviral *Acat2* overexpression in the liver may be a potential therapeutic tool in the treatment of obesity and hypercholesterolaemia.

**Graphical abstract:**

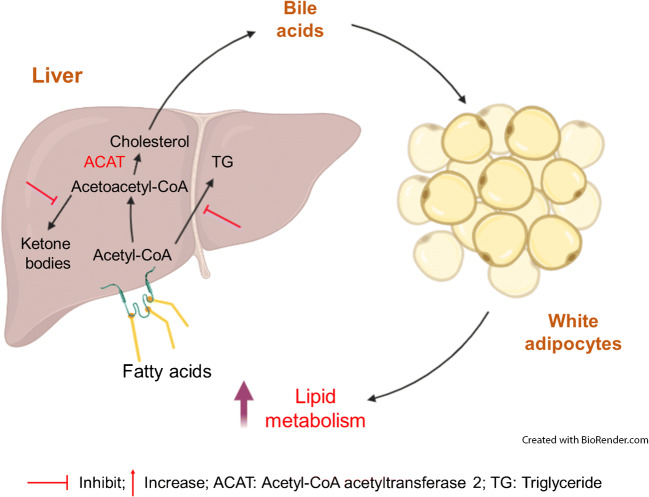

**Supplementary Information:**

The online version contains peer-reviewed and unedited supplementary material available at 10.1007/s00125-022-05829-9.

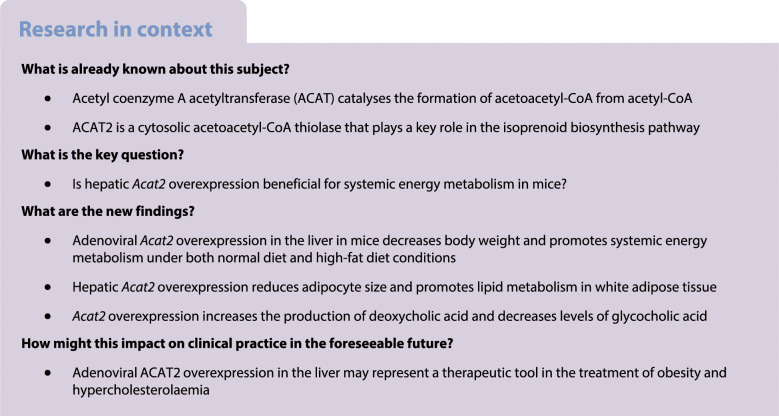



## Introduction

The increasing prevalence of obesity and its associated risks of metabolic diseases and CVD poses a formidable threat to human health [1]. Hypercholesterolaemia is one of the most important risk factors for CVD and is of great concern to the public [2, 3]. Cholesterol is a key component of cell membrane bilayers in higher eukaryotes, and arises from endogenous cholesterol biosynthesis or internalisation of exogenous sources of cholesterol in the form of lipoprotein-cholesterol [4, 5]. Besides its function in maintaining membrane permeability and fluidity, cholesterol modulates functions of membrane proteins and participates in diverse membrane trafficking and transmembrane signalling processes [3, 6, 7]. The de novo synthesis of cholesterol from acetyl-CoA involves multiple-stepped reactions through the mevalonate pathway, with cholesterol being subsequently fatty acylated to form cholesteryl esters or oxidised to form oxysterols in all cell types or to form bile acids and steroid hormones in hepatocytes and steroidogenic cells, respectively [8–10]. These metabolites also play important biological roles either as signal transducers or solubilisers of other lipids [11–13]. Emerging experimental and human evidence has linked altered hepatic cholesterol homeostasis to hypercholesterolaemia and the pathogenesis of CVD [14]. Thus, understanding and targeting cholesterol metabolism in the liver will help develop therapeutical strategies to overcome metabolic disorders and CVD that are associated with hypercholesterolaemia.

Research efforts have been directed towards identifying targets for the treatment of hypercholesterolaemia. Acetyl-CoA acetyltransferase (ACAT), also known as acetoacetyl-CoA thiolase, catalyses the condensation of two molecules of acetyl-CoA to acetoacetyl-CoA, which is the first step in cholesterol biosynthesis [15]. Two ACATs have been identified in humans: cytosolic acetoacetyl-CoA thiolase (encoded by *ACAT2* gene) and mitochondrial acetoacetyl-CoA thiolase (T2, encoded by *ACAT1* gene) [16]. T2, also known as β-ketothiolase, catalyses the synthesis and degradation of ketone bodies [17]. Missense *ACAT1* variants that cause T2 efficiency have been extensively investigated in human diseases [18–20]. However, no genetic approaches have been made to assess the role of ACAT2 in cholesterol homeostasis in vivo.

The preclinical and clinical successes achieved with adeno-associated virus (AAV)-mediated delivery of gene therapies in vivo have helped AAV gain popularity and become the leading platform as an ideal therapeutic vector [21]. Two AAV-based therapeutic agents have been approved by the European Medicines Agency (EMA) and US Food and Drug Administration (FDA). Prominent strategies have also been developed to better confine gene expression to the desired compartment by using tissue- or cell-type-specific promoters [22], including liver-specific gene editing driven by the thyroxine-binding globulin (Tbg) promoter [23, 24]. In the present study, we employed AVV9-mediated hepatic *Acat2* overexpression in mice to access the physiological roles of ACAT2 gain-of-function in liver.

## Methods

For detailed methods, please refer to the electronic supplementary material (ESM) Methods.

### Animal care

Experimental mice used in this study all were of C57BL/6N background and were bred and housed in the animal facility of CAM-SU (Suzhou, China) with free access to water and standard rodent chow food or high-fat diet (HFD; D12451; Research Diets, USA). Mouse maintenance and experimental use were performed according to protocols approved by the CAM-SU Animal Care and Use Committee. Mouse phenotyping experiments were performed by randomly picking mice without noting the exact mouse ear-tag number.

### AAV9 and tail-vein injection

The coding sequence of *Acat2* was retrieved from NCBI (NM_009338) and cloned into GV599 vector (TBGp-MCS-EGFP-3Flag-SV40 PolyA, liver-specific expression driven by a mouse Tbg promoter). The recombinant AAV9 was produced in AAV-293 cells and randomly injected into the tail vein of 8-week-old C57BL/6N mice after purification.

### Indirect calorimetry and body composition measurement

The oxygen consumption ($\dot{V}{\mathrm{O}}_2$) and carbon dioxide production ($\dot{V}{\mathrm{CO}}_2$) of the mice were measured by using an indirect calorimetry system (Oxymax; Columbus Instruments, USA). Total body fat and lean mass in live mice were measured without anaesthesia by using a Minispec LF50 body composition analyser (Bruker, Germany) located in the Small Animal Facility of CAM-SU.

### Treadmill

The $\dot{V}{\mathrm{O}}_2$ and $\dot{V}{\mathrm{CO}}_2$ of mice subjected to treadmill were measured by using a treadmill with indirect calorimetry meter (Oxymax, Columbus Instruments).

### GTT

For the GTT, mice were given an i.p. injection of 100 mg/ml d-glucose (2 g/kg body weight for mice on chow diet) after overnight fasting for 14 h. Tail blood glucose concentrations were measured by a glucometer (Accu-Chek Active; Roche, Switzerland) 15, 30, 60 and 120 min after injection. In the test, mice were caged with blinded cage number in random order.

### Cardiac ultrasonography and ECG

Cardiac ultrasonography was performed using an ultrasound platform incorporated with a probe for mice (VINNO 6, VINNO, China). For ECG, mice were gently removed from their cages and transferred into a ECGenie recording system (Mouse Specifics, USA), which was sized comfortably to accommodate adult mice. Complete results are showed in ESM Tables 1 and 2.

### Blood biochemistry

Blood biochemistry was examined using a clinical chemistry analyser (Hitachi 7100; Hitachi, Japan). Complete results are shown in ESM Table 3.

### H&E staining

Adipose tissues and liver from the control and AAV9-*Acat2* mice were fixed in 4% (wt/vol.) paraformaldehyde for 24 h at room temperature. Then the tissues were embedded in paraffin, blocked and cut at 6 mm. For H&E staining, the sections were deparaffinised, rehydrated and the nuclei stained with haematoxylin for 15 min. Sections were then rinsed in running tap water for 3 min before being stained with eosin for 3 min, then dehydrated and mounted. Images were captured using a Leica DM 6000B fluorescent microscope (Leica, Germany).

### Total RNA extraction and real-time PCR

Total RNA was extracted from cells and tissues by using Trizol reagent (Invitrogen, USA) and then reversed transcribed using random primers and M-MLV reverse transcriptase to make cDNA. Quantitative real-time PCR (qPCR) was carried out with a Lightcycler 480 PCR System (Roche) using SYBR Green Master Mix and gene-specific primers.

### Protein extraction and western blot analysis

Proteins in homogenised liver were analysed by immunoblotting using different antibodies (Anti-GFP, 50430-2-AP and Anti-Beta Tubulin, 10068-1-AP from Proteintech, China; Anti-FLAG, sab4301135 from Sigma, USA).

### Transcriptome sequencing

Total RNA was extracted from liver 3 months after AAV9 injection, and subjected to RNA-seq analysis performed by Azenta Life Sciences (China).

### Non-targeted metabolomics

The non-targeted metabolic profiling analysis was performed by an ultra-HPLC (Vanquish Flex UHPLC system; Thermo Scientific, Bremen, Germany) system coupled with high-resolution MS (Q Exactive Focus; Thermo Scientific).

### Statistical analysis

All analyses were conducted with Student’s *t* test (two-tailed). All experimental data are presented as mean±SEM. Comparisons with *p* values <0.05 were considered statistically significant, and *p* values <0.0, <0.01 and <0.001 are shown.

## Results

### *Acat2* is highly expressed in liver and decreased after HFD-induced obesity

**Fig. 1:**
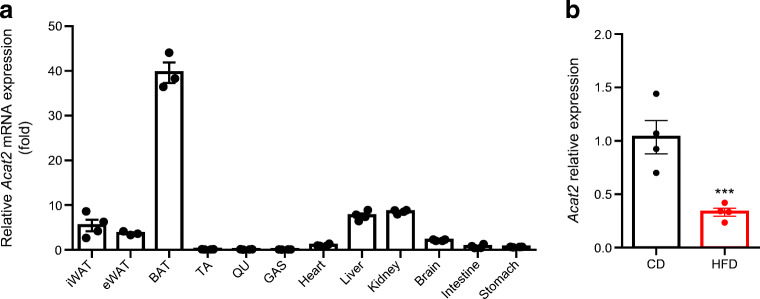
*Acat2* is highly expressed in liver and downregulated in DIO. (**a**) qPCR detection of *Acat2* expression in different mouse tissues (*n*=4). (**b**) Relative levels of *Acat2* in liver from mice fed with HFD or chow diet for 10 weeks (*n*=4). Measured as average of three technical replicates. Data represent mean± SEM. ***p<0.001 (two-tailed *t* test). CD, chow diet; GAS, gastrocnemius; QU, quadriceps; TA, tibialis anterior

We first surveyed the expression of *Acat2* in various mouse tissues. *Acat2* mRNA levels were highest in brown adipose tissue (BAT), followed by lower expression levels in liver and kidney (Fig. 1a). The mRNA levels of *Acat2* were low in muscle tissues (tibialis anterior, quadriceps and gastrocnemius), heart, intestine and stomach (Fig. 1a). As ACATs play a key role in the cholesterol metabolic pathway, we next surveyed the expression level of ACAT2 in liver after diet-induced obesity (DIO). After 10 weeks of HFD feeding, *Acat2* mRNA levels were significantly decreased in liver (Fig. 1b). The results demonstrated that ACAT2-mediated cholesterol metabolism might be inhibited and contribute to the lipid disorder during obesity.

### Adenoviral overexpression of *Acat2* in liver reduces fat mass

**Fig. 2:**
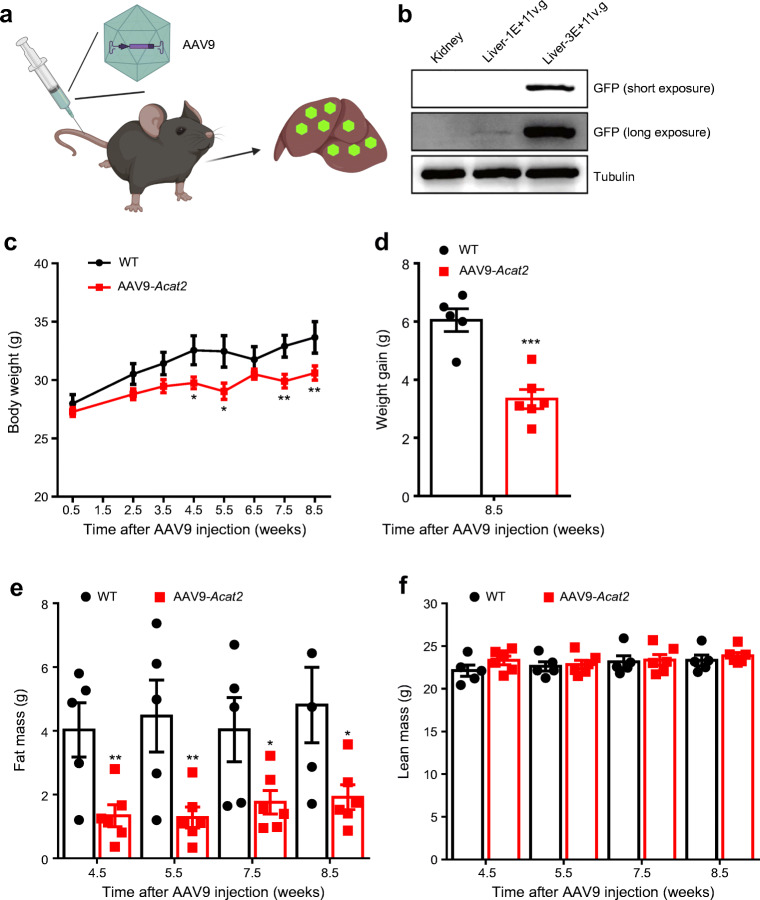
Hepatic *Acat2* overexpression via AAV9 reduces body weight and fat mass of mice. (**a**) Schematic diagram of hepatic *Acat2* overexpression, created with BioRender.com. (**b**) Successful overexpression of ACAT2 in liver but not kidney is shown by GFP western blot. Representative image from three independent experiments. Short exposure: 1s. Long exposure: 120s. (**c**, **d**) Body weight (**c**) and weight gain (**d**) of male mice injected with control and AAV9-*Acat2* virus. (**e**, **f**) Fat mass (**e**) and lean mass (**f**) of WT mice injected with control and AAV9-*Acat2* virus. *n*=5 and 6 control and AAV9-*Acat2* male mice starting from 8 weeks of age, respectively. Data represent mean±SEM. *p<0.05, **p<0.01 and ***p<0.001 (two-tailed *t* test)

We next constructed an adenoviral *Acat2* overexpression system (AAV9*-Acat2*) to achieve liver-specific *Acat2* overexpression. We injected the virus into the tail vein of 6-week-old male mice (Fig. 2a) and specific overexpression was visualised in the liver by GFP western blot 3 weeks after injection. With a virus dose of 3E+11v.g/mouse (where 3E+11v.g=3×10^11^ virus copies) there was only a weak band at 1E+11v.g/mouse (where 1E+11v.g=1×10^11^ virus copies) (Fig. 2b). Consistently, no GFP signal was detected from the kidney, indicating that the virus injection induced liver-specific expression (Fig. 2b).

The body weight of mice injected with AAV9-*Acat2* was significantly reduced from 4.5 weeks after injection and remained low until 8.5 weeks post injection (Fig. 2c). The body weight gain of AAV9-*Acat2*-injected mice was 3.38± 0.89 g, which was lower than the 6.04±0.87 g seen in the control mice (Fig. 2d). The total body fat mass of AAV9-*Acat2* injected mice was >50% smaller than that of the control group from 4.5 weeks to 8.5 weeks post injection (Fig. 2e). There was no difference in lean mass at all tested times when comparing control mice with AAV9-*Acat2*-injected mice (Fig. 2f). These results demonstrate that overexpression of *Acat2* in liver via AAV9 specifically reduces the body fat mass of mice without affecting their lean mass.

### Hepatic *Acat2* overexpression elevates metabolic rate

**Fig. 3:**
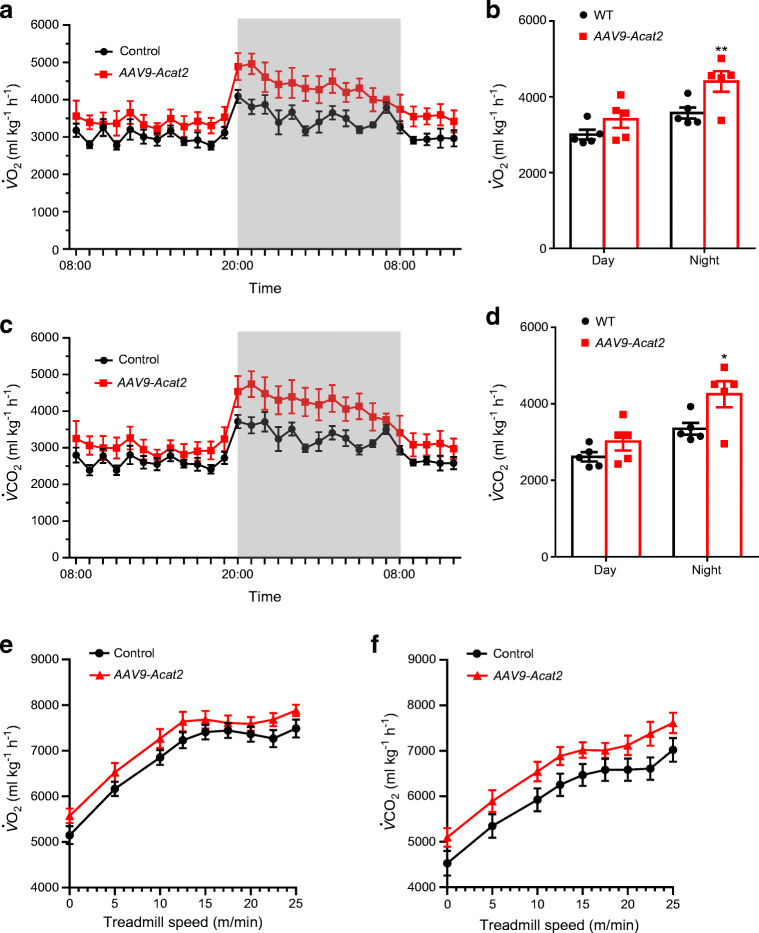
Hepatic *Acat2* overexpression promotes energy expenditure in mice. (**a**–**d**) $\dot{V}{\mathrm{O}}_2$ and $\dot{V}{\mathrm{CO}}_2$ were measured by indirect calorimetry in mice injected with control and AAV9-*Acat2* virus. $\dot{V}{\mathrm{O}}_2$ is shown for a 24 h cycle (**a**) and as an average for day and night (**b**). $\dot{V}{\mathrm{CO}}_2$ is shown for a 24 h cycle (**c**) and as an average for day and night (**d**), calculated from the same dataset. (**e**, **f**) $\dot{V}{\mathrm{O}}_2$ (**e**) and $\dot{V}{\mathrm{CO}}_2$ (**f**) during exercise were measured by a treadmill incorporating indirect calorimetry. *n*=5 and 6 control and AAV9-*Acat2* male mice starting from 8 weeks of age, respectively. Data represent mean±SEM. *p<0.05 and **p<0.01 (two-tailed *t* test)

We next examined how hepatic *Acat2* overexpression affects the systemic metabolism of the mice. Mice injected with AAV9-*Acat2* had higher $\dot{V}{\mathrm{O}}_2$ and $\dot{V}{\mathrm{CO}}_2$ values than the control group (Fig. 3a–d), especially at night when the mice were actively feeding (Fig. 3b,d). The respiratory exchange ratio (RER) did not differ between groups (ESM Fig. 1a,b). Interestingly, the food intake of *Acat2*-overexpressing mice was significantly increased (ESM Fig. 1c). We next ran the mice on a treadmill to measure their metabolic rates during exercise. The results showed that mice injected with AAV9-*Acat2* had higher $\dot{V}{\mathrm{O}}_2$ and $\dot{V}{\mathrm{CO}}_2$ values independently of treadmill speed (Fig. 3e,f). These results collectively suggest that *Acat2* overexpression in liver elevates the metabolic rate of mice and that this effect is independent of muscle metabolism.

### Hepatic *Acat2* overexpression improves glucose tolerance and lowers cholesterol levels

**Fig. 4:**
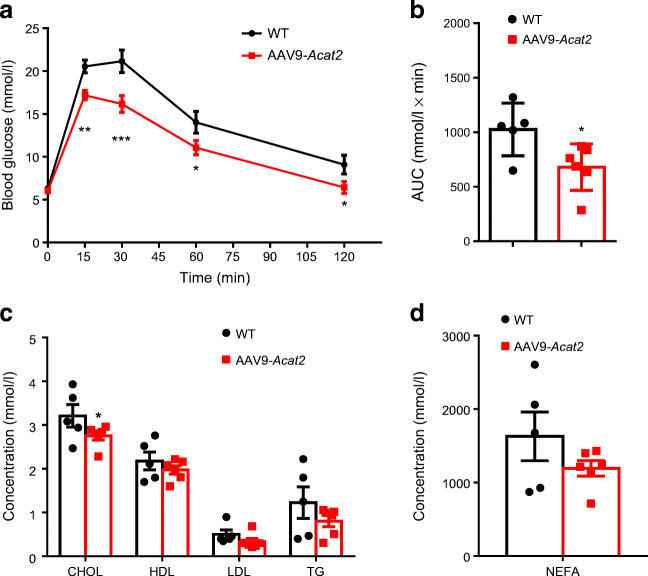
Hepatic *Acat2* overexpression improves glucose tolerance and reduces blood cholesterol levels in mice. (**a**) Blood glucose concentrations during GTT performed in mice 8 weeks after injection of control and AAV9-*Acat2* virus. (**b**) AUC for blood glucose calculated based on data in (**a**). (**c**, **d**) Concentrations of cholesterol, HDL-cholesterol, LDL-cholesterol, TG (**c**) and NEFA (**d**) from the serum of control-virus- and AAV9-*Acat2*-injected mice. *n*=5 and 6 control and AAV9-*Acat2* male mice starting from 8 weeks of age, respectively. Data represent mean±SEM. *p<0.05 and **p<0.01 (two-tailed *t* test). CHOL, cholesterol

We next examined whether *Acat2* overexpression had an impact on systemic glucose and lipid metabolism. In the GTT, *Acat2*-overexpressing mice had lower glucose levels compared with control mice after i.p. injection of glucose (Fig. 4a). Consistently, the AUC of *Acat2*-overexpressing mice was also smaller than that of control mice, suggesting a significantly improved glucose handling ability (Fig. 4b). We next performed blood biochemistry to analyse the serum lipid levels. Serum cholesterol (total) levels were significantly reduced (Fig. 4c) and NEFA showed a non-significant decrease in *Acat2-*overexpressing mice (Fig. 4d). These data indicate that *Acat2* overexpression improves glucose tolerance and decreases serum cholesterol levels in mice.

### Hepatic *Acat2* overexpression inhibits lipid metabolism but promotes the stress response pathway

We further confirmed whether liver and cardiac function were affected by *Acat2* overexpression. Similar to the GTT result, serum glucose levels were not changed in *Acat2*-overexpressing mice compared with control-virus-injected mice (ESM Fig. 2a). Lactate dehydrogenase level was increased in the serum from *Acat2*-overexpressing mice (ESM Fig. 2b). The level of aspartate aminotransferase (AST) in the serum of *Acat2*-overexpressing mice was increased compared with that of the control group but levels of alanine aminotransferase (ALT) and alkaline phosphatase (ALP) were not changed (ESM Fig. 2c). TP (total protein) and ALB (albumin) levels were slightly reduced after AAV9-*Acat2* injection (ESM Fig. 2d).

Heart rate and heart rate variability were not changed by *Acat2* overexpression, as revealed by ECG (ESM Fig. 3a,b). Cardiac ultrasonography showed that *Acat2* overexpression increased the left ventricular internal diameter (LVID) at end-systole but had no effect on the LVID at end-diastole, left ventricular posterior wall (LVPW) at end-diastole, LVPW at end-systole or the total cardiac output (ESM Fig. 3c,d). These results together reveal that hepatic *Acat2* overexpression has minor side-effects on the liver and cardiac function of the mice.

**Fig. 5:**
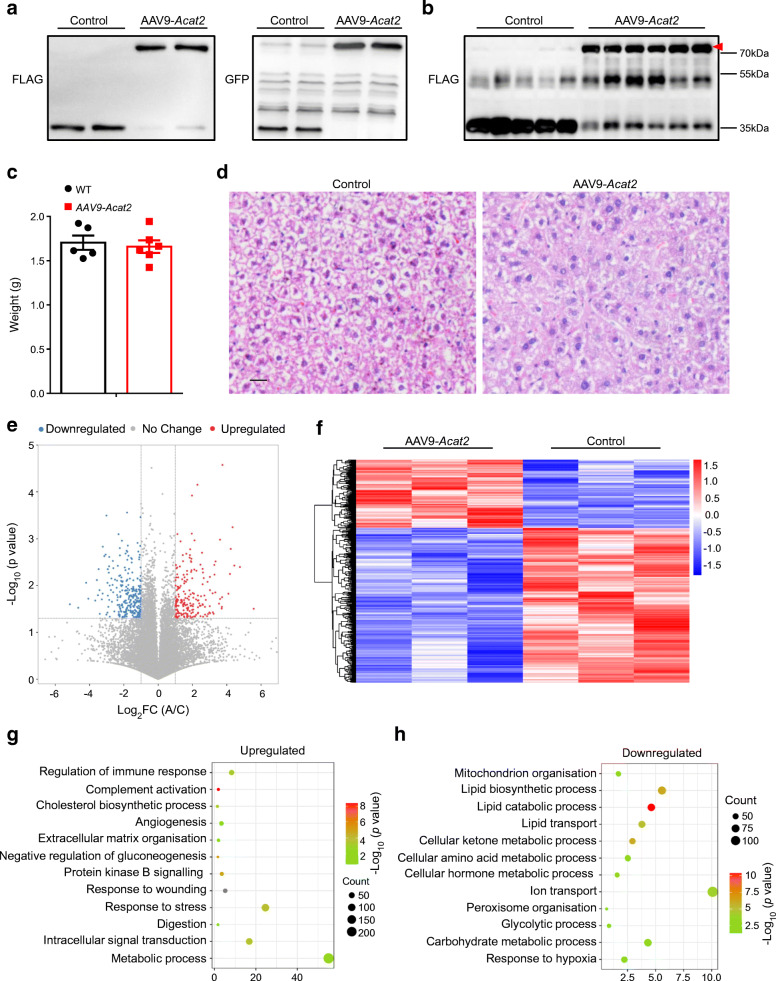
Hepatic *Acat2* overexpression inhibits lipid metabolism pathways in liver. (**a**, **b**) Overexpression of ACAT2 protein in liver of WT mice 3 weeks (**a**) and 3 months (**b**) after AAV9 injection, detected by FLAG and GFP western blot. Representative image from two independent experiments (8 pairs for 3 weeks; 10 control and 11 AAV-*Acat2* for 3 months). (**c**) Weights of liver from control-virus- and AAV9-*Acat2*-injected mice. (**d**) H&E staining of liver from control and AAV9-*Acat2*-injected mice. Scale bar, 50 μm. (**e**) Volcano plot showing DEGs in liver of *Acat2*-overexpressing and control mice. Red dots represent upregulated genes in AAV9-*Acat2-*injected mice and blue dots represent the downregulated genes. (**f**) Heatmap of all the DEGs. (**g**, **h**) GO annotation to identity the key pathways changed in *Acat2-*overexpressing mouse liver. A/C, AAV-ACAT2 group (A) versus control (C); FC, fold change

We then isolated liver from mice injected with AAV9-*Acat2* or control virus. FLAG and GFP western blotting revealed that ACAT2 protein was successfully overexpressed at both 3 weeks and 3 months after AAV9-*Acat2* injection (Fig. 5a,b). *Acat2* overexpression did not affect liver weight (Fig. 5c). H&E staining and lipid quantification both showed that there was less lipid, especially triglyceride (TG), accumulation in the liver after *Acat2* overexpression (Fig. 5d and ESM Fig. 4a). In addition, there was no difference in the content of cholesterol and cholesteryl ester when comparing the two mouse groups (ESM Fig. 4b,c). High-throughput RNA-sequencing was performed to discover differentially expressed genes (DEGs) in the liver of *Acat2-*overexpressing and control mice. After mapping of unique reads and FastQC, we were able to identify a total of 1518 DEGs, of which 1032 were decreased and 486 were increased in *Acat2-*overexpressing mouse liver (Fig. 5e,f and ESM Table 4). Functional annotation and enrichment by using Gene Ontology (GO) revealed a major enrichment of DEGs in the metabolic pathways (Fig. 5g,h and ESM Table 5). Genes involved in mitochondrion organisation (GO: 0007005), lipid catabolic process (GO: 0016042), lipid biosynthetic process (GO: 0008610), lipid transport (GO: 0006869) and carbohydrate metabolic process (GO: 0005975) were all decreased in the AAV9-*Acat2*-injected mice, suggesting an inhibition of lipid and carbohydrate metabolic pathways after *Acat2* overexpression (Fig. 5h). Genes involved in regulation of immune response (GO: 0050776), cholesterol biosynthetic process (GO: 0006695), angiogenesis (GO: 0001525), digestion (GO: 0007586) and response to stress (GO: 0006950) were significantly upregulated in *Acat2*-overexpressing mice (Fig. 5f). These results together demonstrate that *Acat2* overexpression inhibits the expression of genes involved in lipid and carbohydrate metabolism but upregulates genes involved in cholesterol metabolism. In addition, ACAT2 may also participate in the immune response and angiogenesis, thus promoting the stress response pathway.

### Hepatic *Acat2* overexpression causes metabolic remodelling from ketogenesis to the bile acid synthesis pathway

**Fig. 6:**
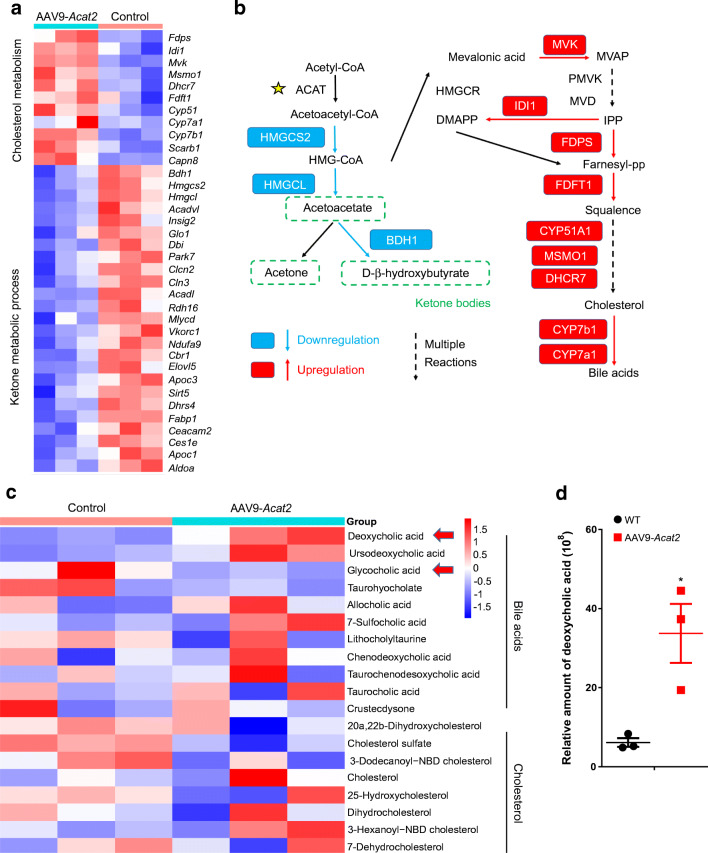
Hepatic *Acat2* overexpression promotes cholesterol metabolism and bile acid biosynthesis pathways in mouse liver. (**a**) Heatmap of DEGs enriched in cholesterol metabolism and ketone metabolic process. (**b**) Pathway showing enzymes involved in cholesterol metabolism and ketone metabolic process; red boxes represent enzymes encoded by upregulated genes and blue boxes represent those encoded by downregulated genes in *Acat2-*overexpressing mouse liver. (**c**, **d**) Heatmap of metabolites of bile acids and cholesterol (**c**), and the relative amount of deoxycholic acid in the liver of control and AAV9-*Acat2-*injected mice. Red arrows indicate two important bile acids. Data represent mean± SEM. *p<0.05 (two-tailed *t* test). DMAPP, dimethylallyl pyrophosphate; Farnesyl-pp, farnesyl-pyrophosphate; IPP, isopentenyl pyrophosphate; MVAP, mevalonatephosphate

ACATs catalyse the formation of acetoacetyl-CoA from acetyl-CoA. Acetoacetyl-CoA can subsequently be used by hydroxymethylglutaryl coenzyme A synthases (HMGCSs) for ketogenesis or de novo cholesterol synthesis [25]. Surprisingly, expression levels of genes involved in ketogenesis, especially genes encoding rate-limiting enzymes (*Hmgcs2*, *Hmgcl* and *Bdh1*), were downregulated after *Acat2* overexpression (Fig. 6a). Cholesterol biosynthesis-related genes, such as *Mvk*, *Idi1*, *Fdps*, *Fdft1*, *Cyp51a1*, *Msmo1* and *Dhcr7* were upregulated (Fig. 6a). Intriguingly, the mRNA levels of key enzymes, *Cyp7a1* and *Cyp7b1*, which catalyse bile acid production, were all upregulated in the *Acat2*-overexpressing liver (Fig. 6a). The results indicate a specific metabolic remodelling in liver by *Acat2* overexpression towards utilisation of acetyl-CoA for bile acid synthesis instead of TG synthesis or ketogenesis (Fig. 6b).

We then performed non-targeted metabolomics to identify differential metabolites in liver of control and *Acat2*-overexpressing mice. Sixty-one differential metabolites were identified, of which 19 were upregulated and 42 were downregulated after *Acat2* overexpression (ESM Fig. 5a, ESM Table 6). Kyoto Encyclopedia of Genes and Genomes (KEGG) pathway enrichment revealed that the most significantly changed pathway was that of ABC transporter (mmu02010), which contained l-glutamic acid, glutathione, l-serine, choline, *N*-acetyl-d-glucosamine, adenosine, taurine, inosine and deoxyuridine (ESM Fig. 5b,c, ESM Table 7). The most abundant changed pathway was alanine, aspartate and glutamate metabolism (mmu00250), including l-glutamic acid, l-asparagine and glucosamine 6-phosphate (ESM Fig. 5b,c, ESM Table 7). Consistent with the gene expression results, metabolites involved in bile secretion (mmu04976) were also significantly changed after *Acat2* overexpression (Fig. 6c and ESM Fig. 5b,c); two metabolites were upregulated (deoxycholic acid and lamivudine) and three were downregulated (glutathione, choline and glycocholic acid). The abundance of deoxycholic acid was increased over fivefold in *Acat2*-overexpressing liver (Fig. 6d). Pathway analysis revealed that bile acids secreted into the bile canaliculus were significantly increased (ESM Fig. 6a). However, the expression levels of genes encoding key bile transporters (*Abcb11* and *Abcc2*) were not changed (ESM Fig. 6b). Taken together, hepatic *Acat2* overexpression changes the composition of secreted bile, in particular increasing the abundance of deoxycholic acid.

### *Acat2* overexpression reduces white adipose tissue mass and promotes lipid metabolism gene expression

**Fig. 7:**
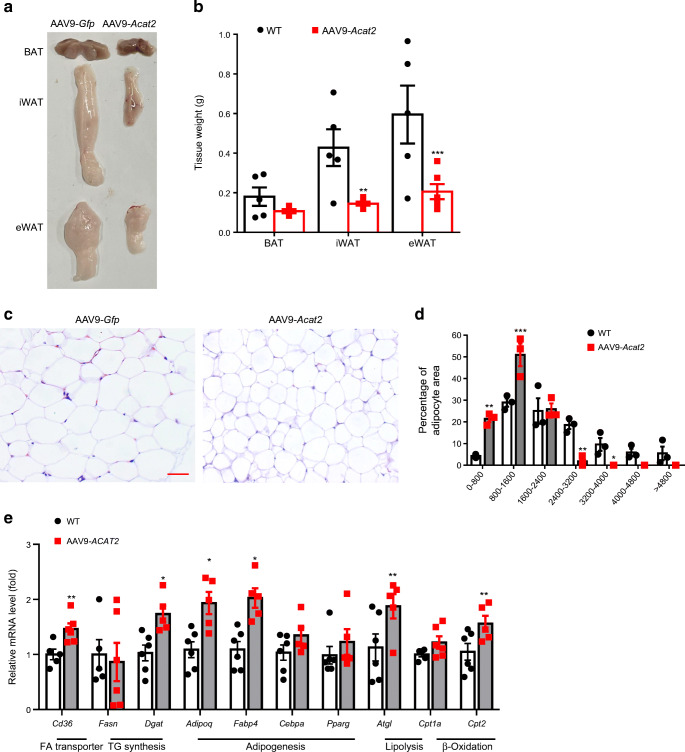
Hepatic *Acat2* overexpression decreases adipocyte size and promotes lipid metabolism in WAT. (**a**, **b**) Representative image (**a**) and weights (**b**) of BAT and WAT (eWAT, and iWAT) depots. *n*=5 and 6 control and AAV9-*Acat2*-injected male mice starting from 8 weeks of age, respectively. (**c**) H&E staining of eWAT from control and AAV9-*Acat2*-injected mice. Scale bar, 50 μm. (**d**) Distribution of adipocyte areas of eWAT from control and AAV9-*Acat2-*injected mice, calculated by averaging 100 adipocytes per image, three images per mouse. (**e**) Relative levels of genes in eWAT involved in fatty acid transport, TG synthesis, adipogenesis, lipolysis and β-oxidation. Measured as the average of three technical replicates. Data represent mean±SEM. *p<0.05, **p<0.01 and ***p<0.001 (two-tailed *t* test). FA, fatty acid

To determine how *Acat2* overexpression reduced the total fat mass, we inspected various fat depots from AAV9-*Acat2*-injected and control mice. The white adipose tissue (WAT) masses were dramatically reduced in mice with hepatic *Acat2* overexpression when compared with control mice, while there was no significant difference in BAT mass (Fig. 7a,b). H&E staining showed that the average adipocyte size was smaller in epididymal WAT (WAT) in the AAV9-*Acat2* group than in the control group (Fig. 7c,d). We then profiled mRNA levels of genes involved in fatty acid transport, TG synthesis, adipogenesis, lipolysis, β-oxidation and browning. The expression levels of *Cd36*, *Dgat*, *Adipoq*, *Fabp4*, *Atgl* and *Cpt2* were significantly increased in eWAT of *Acat2* overexpressed than control mice (Fig. 7e). However, no significant changes in thermogenic and mitochondria-related genes were detected in BAT or inguinal WAT (iWAT) (ESM Fig. 7a,b). Therefore, *Acat2* overexpression promotes lipid metabolism in eWAT.

### Hepatic *Acat2* overexpression protects mice from HFD-induced weight gain and metabolic defects

**Fig. 8:**
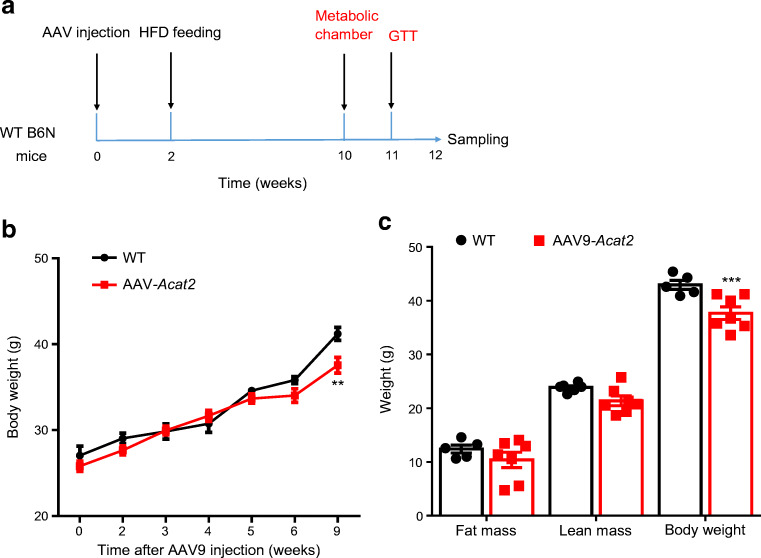
Hepatic *Acat2* overexpression protects mice from body weight gain during HFD feeding. (**a**) Flow chart showing the timing of the AAV9-*Acat2* injection, HFD feeding and sampling. Red text indicates the measurements. (**b**) Body weight of male mice injected with control and AAV9-*Acat2* virus during 9 weeks of HFD. (**c**) Body composition of the mice after 9 weeks of HFD feeding. Data represent mean±SEM. **p<0.01 and ***p<0.001 (two-tailed *t* test)

The phenotype of the AAV9-*Acat2*-injected mice prompted us to investigate the effect of hepatic *Acat2* overexpression on DIO. We injected control or AAV9-*Acat2* virus into wild-type (WT) mice 2 weeks before switching them to HFD (45%) (Fig. 8a). The body weight of the two groups of mice started to show a difference after 6 weeks of HFD feeding, and at 7 and 10 weeks the weight of the AAV9-*Acat2*-injected mice was significantly lower than that of the control mice (Fig. 8b,c). Body composition analysis showed a decrease in both fat mass and lean mass during the HFD feeding but the difference was not statistically significant (Fig. 8c).

**Fig. 9:**
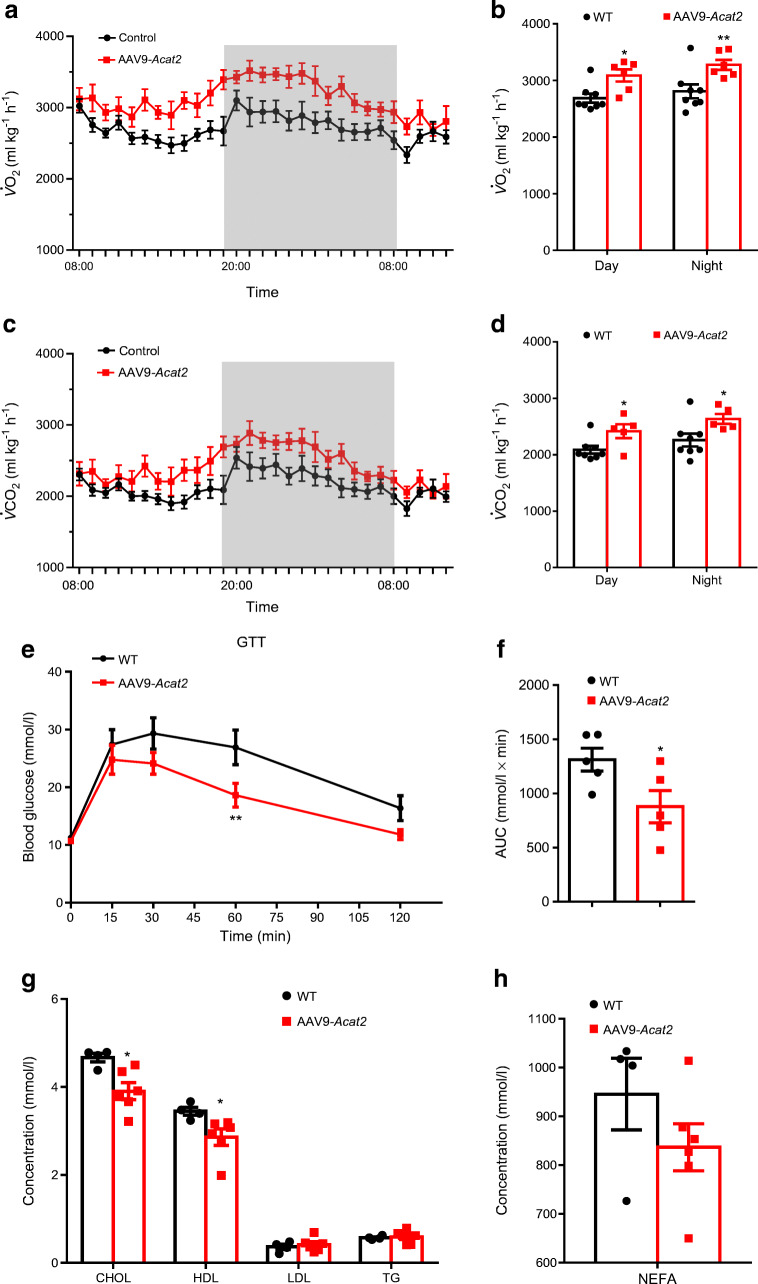
Hepatic *Acat2* overexpression elevates systemic energy metabolism and reduces blood cholesterol levels in mice after HFD feeding. (**a**–**d**) WT mice were injected with control and AAV9-*Acat2* virus after 8 weeks of HFD feeding. $\dot{V}{\mathrm{O}}_2$ and $\dot{V}{\mathrm{CO}}_2$ were measured by indirect calorimetry. $\dot{V}{\mathrm{O}}_2$ is shown for a 24 h cycle (**a**) and as an average for day and night (**b**). $\dot{V}{\mathrm{CO}}_2$ is shown for a 24 h cycle (**c**) and as an average for day and night (**d**), calculated from the same dataset. (**e**) Blood glucose concentrations during a GTT performed on mice after 9 weeks of HFD feeding. (**f**) AUC for blood glucose was calculated based on data in (**e**). (**g**, **h**) Concentrations of cholesterol, HDL-cholesterol, LDL-cholesterol, TG (**g**) and NEFA (**h**) in the serum of control and AAV9-*Acat2*-injected mice after 10 weeks of HFD feeding. *n*=4 and 6 control and AAV9-*Acat2* male mice, respectively. Data represent mean±SEM. *p<0.05 and **p<0.01 (two-tailed *t* test). CHOL, cholesterol

Consistently, mice injected with AAV9-*Acat2* had higher $\dot{V}{\mathrm{O}}_2$ and $\dot{V}{\mathrm{CO}}_2$ during both day and night compared with the control group under HFD feeding (Fig. 9a–d). The RER did not differ between the groups (ESM Fig. 8a,b). We also tested the glucose tolerance of the mice. The *Acat2*-overexpressing mice fed with HFD exhibited improved glucose tolerance when compared with control mice (Fig. 9e,f). In addition, concentrations of serum cholesterol (total) and HDL-cholesterol were also significantly decreased in *Acat2*-overexpressing mice after HFD feeding (Fig. 9g). Levels of TG, LDL-cholesterol and NEFA showed no difference between the groups (Fig. 9g,h and ESM Table 8). Interestingly, the levels of ALT were significantly decreased in the serum of *Acat2*-overexpressing mice compared with control mice, while levels of AST, TP and ALB were not changed (ESM Fig. 9a,b and ESM Table 8). Taken together, hepatic *Acat2* overexpression elevates the metabolic rate and protects mice from HFD-induced glucose intolerance and hypercholesterolaemia.

## Discussion

**Fig. 10:**
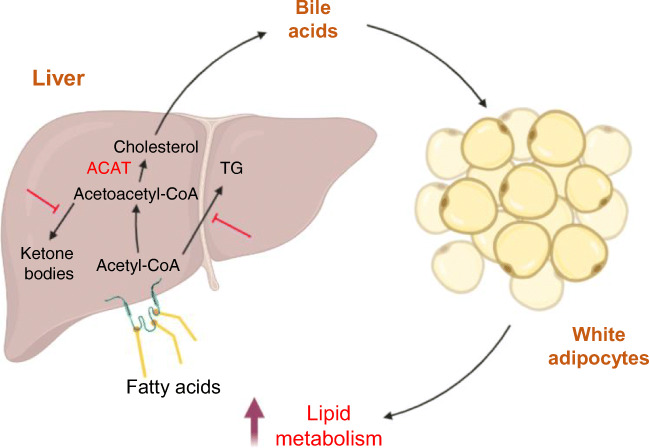
A model depicting hepatic *Acat2* overexpression in vivo, created with BioRender.com

Our study demonstrates a previously unrevealed role for hepatic *Acat2* overexpression in weight control through boosting the metabolic rate. Adenoviral *Acat2* overexpression reduced body weight by lowering total fat mass without affecting lean mass. *Acat2*-overexpressing mice displayed higher $\dot{V}{\mathrm{O}}_2$ and $\dot{V}{\mathrm{CO}}_2$ in normal conditions and during exercise. *Acat2* overexpression promoted glucose clearance and lowered serum cholesterol levels, possibly through enhancing production of bile acids (especially deoxycholic acid) in the liver. In addition, *Acat2*-overexpressing mice gained less body weight, had a higher metabolic rate after HFD feeding and were protected from HFD-induced glucose intolerance and hypercholesterolaemia. Hepatic *Acat2* overexpression inhibited TG, glucose and ketone body metabolism pathways in the liver but promoted lipid metabolism in WAT (Fig. 10). Thus, as the *Acat2* level in liver is decreased during HFD-induced obesity, our results suggest that liver-targeted adenoviral *Acat2* overexpression represents a potential therapeutic strategy for obesity and its associated hypercholesterolaemia.

ACATs catalyses the conversion of acetyl-CoA to acetoacetyl-CoA, which subsequently enters the ketogenesis and multi-stepped cholesterol biosynthesis pathways [26, 27]. ACAT1 is localised in mitochondria and is involved in ketogenesis, and its mutation has been reported to cause diseases [16]. Less is known about ACAT2, except for its role in cytosolic acetoacetyl-CoA production, without data coming from gain-of-function and loss-of-function studies using genetic tools. In this study, we found that *Acat2* was decreased in the liver of HFD-induced obese mice, prompting us to explore whether hepatic *Acat2* overexpression is beneficial for lowering lipid levels and promoting systemic metabolism. This first *Acat2* gain-of-function study clearly showed positive results and potential clinical application, though the current experiments were based on WT mice under normal diet or HFD. Further studies conducted on different mouse models should be performed, including high-cholesterol diet, *ob*/*ob*, *db*/*db* and LDL-cholesterol-receptor knockout mice (hypercholesterolaemia), to investigate the effects of hepatic *Acat2* overexpression in metabolic disorders.

We evaluated the liver and heart function of *Acat2*-overexpressing mice and found that the AST level was increased, in excess of the normal range of C57B6N mice [28]. This indicates that *Acat2* overexpression may cause liver stress or inflammatory responses. Supporting this, RNA-seq data revealed that genes involved in the stress response and innate immune response were upregulated. Since AAVs have emerged as effective and safe tools for in vivo gene delivery, we believe that the elevated serum AST could be a consequence of ACAT2-mediated changes in the lipid metabolism of liver. It has been extensively reported that lipid metabolic pathways are closely associated with chronic hepatic inflammation [29, 30]. For instance, the Gram-positive bacteria binding receptor TLR2, which can also bind dietary fatty acids and plays a role in the progression of the metabolic syndrome [31–33], was upregulated in *Acat2-*overexpressing liver. To our surprise, the elevated levels of AST were diminished after HFD feeding, while ALT levels were decreased, suggesting that *Acat2* overexpression may protect mice from liver damage in DIO. In addition, cardiac ultrasonography and ECG showed *Acat2* overexpression to have mild effects, with a slight increase in the LVID at end-systole but no impact on other tested indexes, especially the ejection fraction. However, future studies should put more effort into monitoring the long-term liver and heart function in *Acat2*-overexpressing mice.

An intriguing observation in our present study was that *Acat2* overexpression inhibited glycolytic, TG synthesis, mitochondrial-related and ketone body metabolic pathways but upregulated genes involved in cholesterol metabolism, especially the bile acid biosynthesis pathway. Bile acids are the end-products of cholesterol, serving as important physiological agents in nutrient absorption and glucose, lipid and energy metabolism control [34–36]. The expression levels of key enzymes in bile acid synthesis pathways, CYP7A1 and CYP7B1 [37, 38], are increased after *Acat2* overexpression. We also found that the food intake of *Acat2*-overexpressing mice was significantly increased. Similar results have been reported in mice lacking *Cyp8b1*, which disrupts bile acid composition and lowers food intake [39]. Besides, dietary bile acid supplements were found to enhance energy expenditure and protect mice from DIO [35, 36], consistent with our own findings. However, we detected improved lipid metabolism in eWAT but did not observed any changes in the thermogenic gene expression in BAT and iWAT, findings that are inconsistent with chenodeoxycholic acid treatment [40]. Indeed, we detected a dramatic increment in the concentrations of deoxycholic acid as well as bile secretion into the bile canaliculus. It is worth mentioning that an injectable synthetic form of deoxycholic acid was approved by the FDA in 2016 for reduction of fat under the chin [41, 42]. Bile acids exert beneficial effects on glucose metabolism [43] and increased serum deoxycholic acid concentration is also significantly associated with decreased fasting blood glucose and metabolic improvement in individuals with type 2 diabetes who are treated with saxagliptin [44]. Another bile acid, glycocholic acid, which is reported to be dramatically increased upon liver injury and liver disease [45], was found to be decreased in our mice overexpressing *Acat2* in the liver. While deoxycholic acid concentrations are negatively associated with liver injury and liver disease [45], in individuals with non-alcoholic steatohepatitis (NASH), bile acid concentrations are higher and their composition is altered in liver tissue when compared with liver from disease-free individuals [46, 47]. Thus, the altered bile acid pool and composition in *Acat2*-overexpressing liver may be responsible for the improved metabolism in hepatic-*Acat2*-overexpressing mice.

*Acat2* overexpression provides a potential therapeutic strategy for obesity and hypercholesterolaemia, yet the current methods and results are limited. Even though we achieved liver-specific *Acat2* overexpression and observed very promising phenotypes by using AAV9-mediated gene delivery, the dose of injection, duration of expression period and t½ of overexpressed protein remain unclear. Besides expanding the experiments to cover different disease models, future studies should be concerned with discovering the mechanisms upstream of *Acat2* that lead to its suppression of DIO. On the other hand, efforts should be focused on developing new *Acat2* overexpression strategies, especially those utilising controllable genetic manipulation (e.g. the tetracycline-inducible [Tet-On or Tet-Off] or doxycycline-inducible systems) to control *Acat2* overexpression [48, 49]. Besides, it is exciting to take advantage of the recent mRNA modification and delivery tools, which have been widely used as mRNA vaccines during the coronavirus disease 2019 (COVID-19) pandemic worldwide [50]. Nanoparticles that encapsulate modified *ACAT2* mRNA for targeted liver delivery with proper release speed are ideal methods for the future.

## Supplementary Information

ESM Figures (1.20 MB)

ESM Tables (6.20 MB)

## Data Availability

All data generated or analysed during this study are included in this published article (and its ESM).

## Abbreviations

AAV: Adeno-associated virus
ACAT: Acetyl-CoA acetyltransferase
ALB: Albumin
ALP: Alkaline phosphatase
ALT: Alanine aminotransferase
AST: Aspartate aminotransferase
BAT: Brown adipose tissue
DEG: Differentially expressed gene
DIO: Diet-induced obesity
eWAT: Epididymal WAT
FDA: Food and Drug Administration
GO: Gene Ontology
HFD: High-fat diet
iWAT: Inguinal WAT
LVID: Left ventricular internal diameter
LVPW: Left ventricular posterior wall
RER: Respiratory exchange ratio
Tbg: Thyroxine-binding globulin
TP: Total protein
TG: Triglyceride
WAT: White adipose tissue
WT: Wild-type
