# Supplementary material for: Hepatic Acat2 overexpression promotes systemic cholesterol metabolism and adipose lipid metabolism in mice
Source: Diabetologia. 2022 Nov 15;66(2):390–405. doi: 10.1007/s00125-022-05829-9 (PMC9665029; doi:10.1007/s00125-022-05829-9)
Supplement: Supplementary file 1 — (1.20 MB) [file 125_2022_5829_MOESM1_ESM.pdf]

Electronic Supplementary Material

ESM Methods

Animal care

Experimental mice used in this study all were of C57BL/6N background and were breaded and housed in the animal facility of CAM-SU (Suzhou, China) with free access to water and standard rodent chow food or high-fat diet (HFD; D12451; Research Diets, USA). Mouse maintenance and experimental use were performed according to protocols approved by the CAM-SU Animal Care and Use Committee.

Adeno-associated virus 9 (AAV9) and tail vein injection

AAV9 particles were concentrated by centrifugation and filtration to a final concentration of ~5E+13v.g./mL (GeneChem, China). Control AAV9 was made in the same process by using empty GV599 vector. For *Acat2* overexpression, 100μL of AAV9-*Acat2* or control AAV9 (Total of 3E+11v.g., diluted in saline) was randomly injected into the tail vein of 8-week-old C57BL/6N.

Indirect calorimetry and body composition measurement

The Oxygen consumption (VO_2) and carbon dioxide production (VCO_2) of the mice were measured by using an indirect calorimetry system (Oxymax, Columbus Instruments). The system was kept in a stable environmental temperature (24 °C) that also had a 12-h light (8 AM-8 PM), 12-h dark cycle (8 PM-8 AM). Mice were individually placed in each chamber with free access to food and water. Mice were adapted to the chamber for 24 h before the measurements. The data were presented as uncorrected energy expenditure levels. Average energy expenditure of day (8 AM-8 PM) and night (8 PM-8 AM) values were the average mean value of all points measured during the 12h period.

Total body fat and lean mass in live animals without anesthesia were measured by using a Minispec LF50 body composition analyzer located in Small Animal Facility of CAM-SU. Animals were placed in a specially sized, clear plastic holder without sedation or anesthesia. The holder was then inserted into a tubular space in the side of the Minispec LF50 system. The animals were forced to not move in the holder to ganrantee the accuracy of result. Each scan took about 2 minutes.

Treadmill

Mice were firstly trained started 5 days before testing at a speed of 5m/min for 5 min to adapted to the treadmill. Mice were forced to run with an electric shock setting at constant 0.7mA on a 15% incline. Then on the day of experiment,run the indirect calorimetry program and the treadmill program at the same time. Mice were allowed to run at a constant speed (5m/min) for 5 min, before increasing the speed at a rate of 2.5 m/min every 2 min, then the mice were running at 25 m/min for the next 4min. After 25 minutes, stop the treadmill program and the indirect calorimetry program. Then remove the mice and clean the treadmill with 75% alcohol.

Cardiac ultrasonography and electrocardiogram

Cardiac ultrasonography was visualized using an ultrasound platform incorporated with a probe for mice (VINNO 6, VINNO). Mice were anesthetized in the induction chamber before sedated onto the operating board. Then a nose cone was applied to ensure anesthesia and hair removal cream was utilized to remove fur in the chest area. Wipe with wet gauze to ensure all hair was removed. Move the animal to the imaging platform with a heatpad to maintain body temperature at 36-37 °C. During imaging, reduce anesthesia to maintain proper heart rate. If the animal shows signs of being awake, use a higher concentration of anesthetic. Then gel was applied on to the imaging area and probe was made contacted to the gel until it was fully covered. The image of the heart was then taken in the short-axis mode with papillary muscles being the point of reference. The papillary muscles were adjusted paralleled to the screen in B-Mode, then pressed the M-mode button and placed the yellow line in the middle of the LV. Change the Display window to 1000ms. If image was not clear, move the yellow line to the left or right. Or left the line in the center of the LV and moved the platform left or right, forward or backwards. Once the imaging was complete, removed animal from the platform and allowed them to recover on a heating pad. Complete results were showed in Table S3.

For electrocardiogram (ECG), mice were gently removed from their own cages and transferred into a ECGenie™ recording system (Mouse Specifics, Inc., USA) that sized comfortably to accommodate adult mice. A pair of ECG electrodes (silver-chloride) were embedded in the floor of the enclosure and spaced to provide contact between the

electrodes and animals' paws. Since even modest handling of mice may induce alterations in heart rate, each mouse was permitted to acclimatize for ∼10 min prior to collection of data. The signals were digitized at a sampling rate of 2000 samples/s. When mice were positioned such that a forepaw and hind paw were not uniquely in contact with one of the electrodes, the output from the amplifier was discarded. Only data from continuous recordings were used in the analyses. Each signal was analyzed using e-MOUSE™, which incorporates Fourier analyses and linear time-invariant digital filtering of frequencies below 3 Hz and above 100 Hz to minimize environmental signal disturbances.

Blood biochemistry

Blood biochemistry was performed by using a clinical chemistry analyzer (Hitachi 7100). Appropriate volume of blood that required (160-200μl of plasma) for test was collected from each mouse, and transfer to gel tube containing lithium Heparin. Centrifuge for 15 minutes at 5000 rpm in a refrigerated centrifuge set at 4°C. If plasma samples cannot be analyzed immediately, keep them in -80°C before analysis. Use plasma samples undiluted or diluted to a ratio of 1:2 with deionized water if the volume was insufficient.

H&E staining

Adipose tissues and liver from the control and AAV9-Acat2 mice were fixed in 4% paraformaldehyde (PFA , wt/vol)for 24 h at room temperature. Then the tissues were embedded in paraffin, blocked and cut at 6 mm. For H&E staining, the sections were deparaffinised, rehydrated and the nuclei stained with haematoxylin for 15 min. Sections were then rinsed in running tap water for 3 min before being stained with eosin for 3 min, then dehydrated and mounted. Images were captured using a Leica DM 6000B fluorescent microscope. (Leica, Germany).

Lipid measurement in liver

Total TG, cholesterol and CE were extracted from liver samples of AAV9-*Acat2* or control AAV9 mice, and measured by total TG kit (NJJCBIO, a110-1-1), total cholesterol kit (NJJCBIO, a111-1-1), and mouse CE kit (Sino Best, YX-030500M) according to the manufacture's protocols, separately.

Total RNA extraction and real-time PCR

Total RNA was extracted from cells or tissues by using Trizol Reagent according to the manufacturer's instructions. The purity and concentration of the extracted RNA were measured by a spectrophotometer (Nanodrop 3000, Thermo Fisher) at 260 and 280 nm. Ratios of absorption (260/280 nm) of all samples were made sure to be ~2.0. 3 μg of RNA were reversed transcribed using random primers and M-MLV reverse transcriptase to make cDNA. Real-time PCR was carried out with a Roche Lightcycler 480 PCR System using SYBR Green Master Mix and gene-specific primers. Primer sequences were retrieved from PrimerBank and listed below in Primer Table. The $2^{-\Delta\Delta CT}$ method was used to analyze the relative changes in gene expression normalized against mouse β-Actin as internal control.

Primer Table

Primer	Sequence (5'—3')
*qAcat2*_F	CCCGTGGTCATCGTCTCAG
*qAcat2*_R	GGACAGGGCACCATTGAAGG
*qCpt1a*_F	CTCCGCCTGAGCCATGAAG
*qCpt1a*_R	CACCAGTGATGATGCCATTCT
*qCpt2*_F	CAGCACAGCATCGTACCCA
*qCpt2*_R	TCCCAATGCCGTTCTCAAAAT
*qCd36*_F	ATGGGCTGTGATCGGAACTG
*qCd36*_R	GTCTTCCCAATAAGCATGTCTCC
*qFasn*_F	GGAGGTGGTGATAGCCGGTAT
*qFasn*_R	TGGGTAATCCATAGAGCCCAG
*qDgat*_F	TCCGTCCAGGGTGGTAGTG
qDgat_R	TGAACAAAGAATCTTGCAGACGA
*qAdipoq*_F	TGTTCCTCTTAATCCTGCCCA
*qAdipoq*_R	CCAACCTGCACAAGTTCCCTT
*qFabp4*_F	AAGGTGAAGAGCATCATAACCCT
*qFabp4*_R	TCACGCCTTTCATAACACATTCC
*qC/EBPα*_F	CAAGAACAGCAACGAGTACCG
*qC/EBPα*_R	GTCACTGGTCAACTCCAGCAC
*qPparγ*_F	TCGCTGATGCACTGCCTATG
*qPparγ*_R	GAGAGGTCCACAGAGCTGATT
*qAtgl*_F	CTGAGAATCACCATTCCCACATC
*qAtgl*_R	CACAGCATGTAAGGGGGAGA

*qUcp1*_F	AGGCTTCCAGTACCATTAGGT
*qUcp1*_R	CTGAGTGAGGCAAAGCTGATTT
*qPrdm16*_F	CCACCAGCGAGGACTTCAC
*qPrdm16*_R	CCACCAGCGAGGACTTCAC
*qCidea*_F	TGACATTCATGGGATTGCAGAC
*qCidea*_R	GGCCAGTTGTGATGACTAAGAC

Protein extraction and western blot analysis

Protein was extracted from homogenized liver samples with RIPA buffer (150 mM NaCl, 1% NP-40, 0.5% sodium Deoxycholate, 0.1% SDS, 50 mM Tris-HCl, pH 8.0) that contained a protease inhibitor cocktail (Sigma) and phosphatase inhibitors NaF and Na_3VO_4. Protein concentrations were determined using Pierce BCA Protein Assay Reagent (Pierce Biotechnology). Equal amount of protein from each sample was loaded for electrophoresis (Bio-Rad). Proteins were separated by SDS–PAGE, transferred to a polyvinylidene fluoride membrane (Millipore Corporation), incubated in blocking buffer (5% fat-free milk in TBS) for 1 h at room temperature (RT), then incubated with primary antibodies (Anti-GFP, 50430-2-AP, 1:1000 in 5% fat-free milk in TBS and Anti-Beta Tubulin, 10068-1-AP from Proteintech, China, 1:3000 in 5% fat-free milk in TBS; Anti-FLAG, sab4301135 from SIGMA, USA, 1:3000 in 5% fat-free milk in TBS) in blocking buffer overnight at 4 °C. Then the membrane was incubated with secondary antibody (HRP AffiniPure goat anti-rabbit IgG, 111-035-003 from Jackson ImmunoResearch, USA, 1:10000 in TBST) at room temperature for 1h.

Transcriptome sequencing

Total RNA was extracted from liver after 3-month of AAV9 injection, and subjected to RNA-seq analysis performed by Azenta Life Sciences. Briefly, RNA quality analysis was checked by Agarose Gel Electrophoresis and Agilent 2100. A complementary DNA library was then constructed using mRNA enriched by anti-polyA beads, and sequencing was performed according to the Illumina HiSeq standard protocol. Raw reads from RNA-seq libraries are filtered to remove reads containing adapters or reads of low quality. After filtering, statistics analysis of data production and quality was performed to confirm the

sequencing quality. Reference genome and gene annotation files were downloaded from a genome website browser (NCBI/UCSC/Ensembl). TopHat2 was used for mapping the filtered reads to the reference genome. For the quantification of gene expression level, HTSeq V0.6.1 was used to analyze the read numbers mapped for each gene. The FPKM of each gene was calculated based on the gene read counts mapped to genes or exons. A differential expression analysis was performed using the DESeq R package (1.10.1) with the threshold of significance set as $p < 0.05$. Heatmap was made by an online tool (http://heatmapper.ca/) based on the Log_2FPKM. Gene Ontology annotation was done by using the DAVID Bioinformatics Resources (https://david.ncifcrf.gov/).

Non-targeted metabolomics

The non-targeted metabolic profiling analysis was performed by using an ultra-high performance liquid chromatography (Vanquish Flex UHPLC system, Thermo Scientific, Bremen, Germany) system coupled wth high-resolution mass spectrometry (Q Exactive Focus, Thermo Scientific, Bremen, Germany). The raw data were firstly converted to mzXML format by MSConvert in ProteoWizard software package and processed using XCMS for feature detection, retention time correction and alignment. The metabolites were identified by accuracy mass (< 30 ppm) and MS/MS data which were matched with HMDB[6] (http://www.hmdb.ca), massbank (http://www.massbank.jp/), LipidMaps (http://www.lipidmaps.org), mzclound (https://www.mzcloud.org) and KEGG (http://www.genome.jp/kegg/). The robust LOESS signal correction was applied for data normalization to correct for any systematic bias. After normalization, only ion peaks with relative standard deviations (RSDs) less than 30 % in QC were kept ensuring proper metabolite identification. The Ropls software was used for all multivariate data analyses and modelings. After scaling data, models were built on principal component analysis (PCA), orthogonal partial leastsquare discriminant analysis (PLS-DA) and partial least-square discriminant analysis (OPLS-DA). The metabolic profiles could be visualized as score plot, where each point represents a sample. The corresponding loading plot and S-plot were generated to provide information on the metabolites that influence clustering of the samples. All the models evaluated were tested for over fitting with methods of permutation tests. The descriptive performance of the models was determined by R2X

(cumulative) (perfect model: R2X (cum) = 1) and R2Y (cumulative) (perfect model: R2Y (cum) = 1) values while their prediction performance was measured by Q2 (cumulative) (perfect model: Q2 (cum) = 1) and a permutation test. The permuted model should not be able to predict classes: R2 and Q2 values at the Y-axis intercept must be lower than those of Q2 and the R2 of the non-permuted model. OPLS-DA allowed the determination of discriminating metabolites using the variable importance on projection (VIP). The P value, Variable importance projection (VIP) produced by OPLS-DA, fold change (FC) was applied to discover the contributable-variable for classification. Finally, P value < 0.05 and VIP values > 1 were considered to be statistically significant metabolites.

Differential metabolites were subjected to pathway analysis by MetaboAnalyst, which combines results from powerful pathway enrichment analysis with the pathway topology analysis. The identified metabolites in metabolomics were then mapped to the KEGG pathway for biological interpretation of higher-level systemic functions. The metabolites and corresponding pathways were visualized using KEGG Mapper tool.

Statistical analysis

All analyses were conducted with Student's t test (two-tailed). All experimental data are presented as mean ± SEM. Comparisons with p values <0.05, <0.01 or<0.001 were considered statistically significant.

ESM Figures

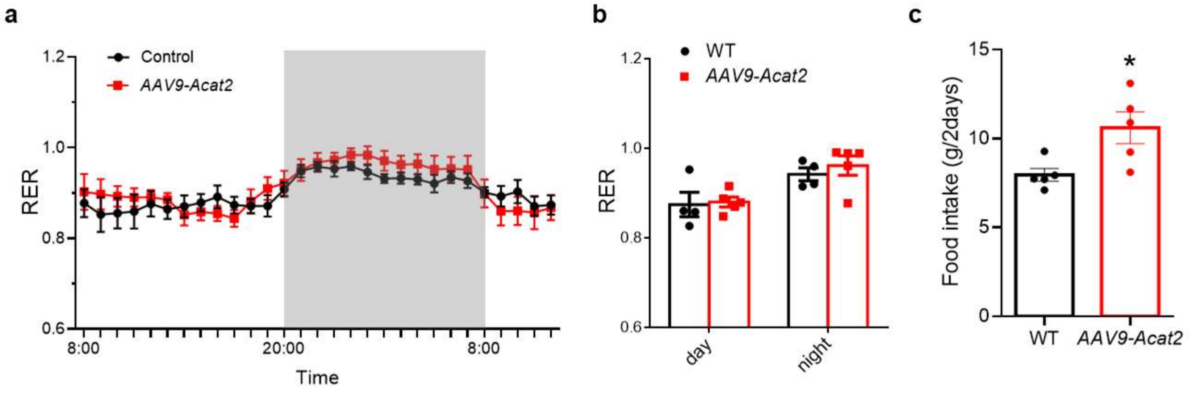

ESM Fig. 1 (a, b) Respiratory exchange rate (RER) is measured by an indirect calorimetry. RER is showed for a 24-hour cycle (a) and average day and night RER (b) are calculated of WT mice injected with control and *AAV9-Acat2* virus. (c) Food intake of the mice as calculated from the metabolic chamber. N=5 and 6 male mice control and *AAV9-Acat2* groups starting from 8-week-old, respectively. Data represent mean±s.e.m. (t-test: * $P<0.05$).

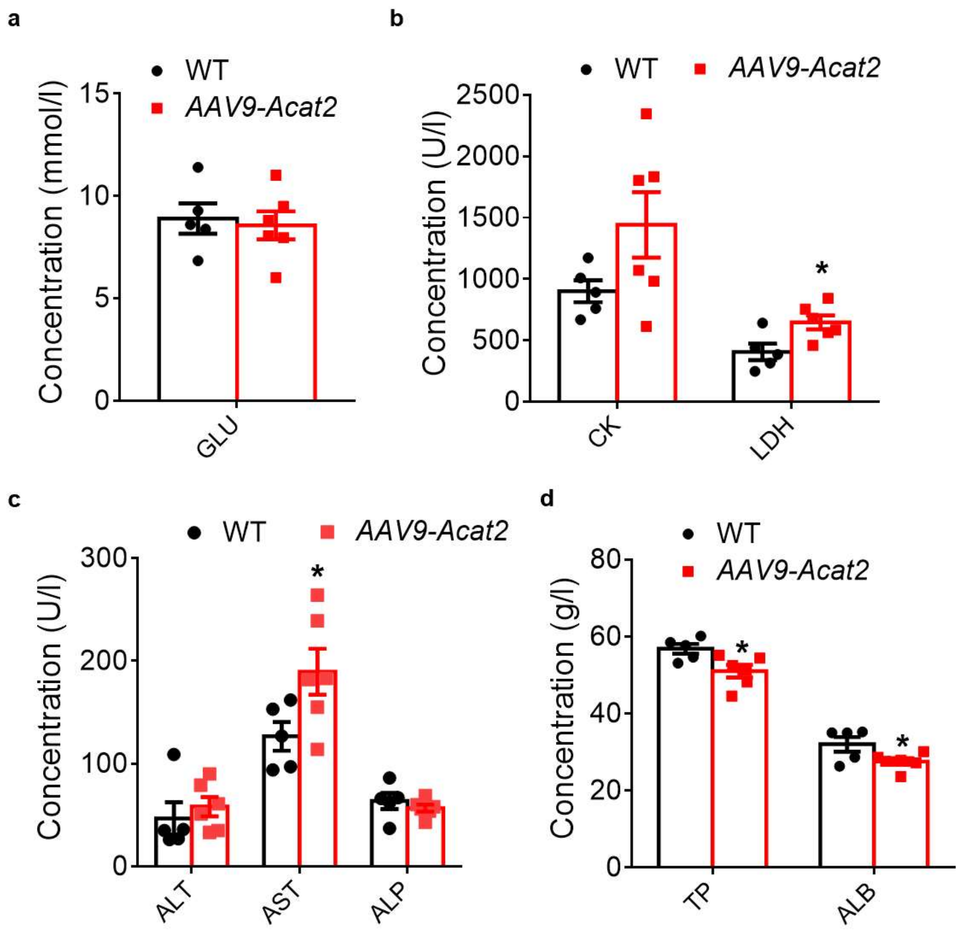

ESM Fig. 2 (a-d) Concentrations of glucose (a), CK and LDH (b), ALT, AST and ALP (c), TP and ALB (d) from the serum of control and *AAV9-Acat2* injected mice. N=5 and 6 male mice control and *AAV9-Acat2* groups starting from 8-week-old, respectively. Data represent mean±s.e.m. (t-test: * $P<0.05$).

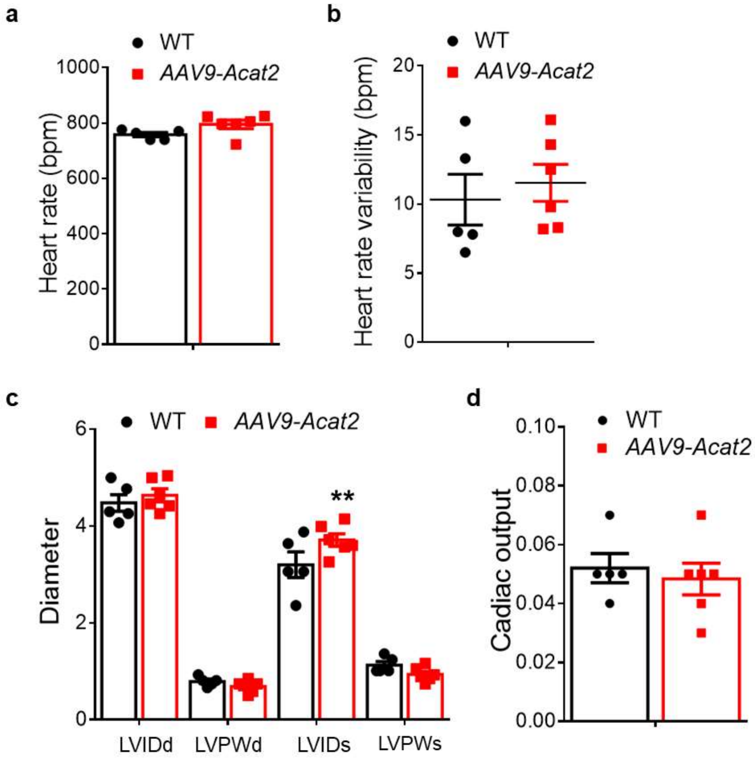

ESM Fig. 3 (a, b) Heart rate (a) and heart rate variability (b) measured by electrocardiogram. (c, d) LVIDd, LVPWd, LVIDs and LVPWs (c) and the total cardiac output (d) measured by cardiac ultrasonography. N=5 and 6 male mice control and *AAV9-Acat2* groups starting from 8-week-old, respectively. Data represent mean±s.e.m. (t-test: ** P<0.01).

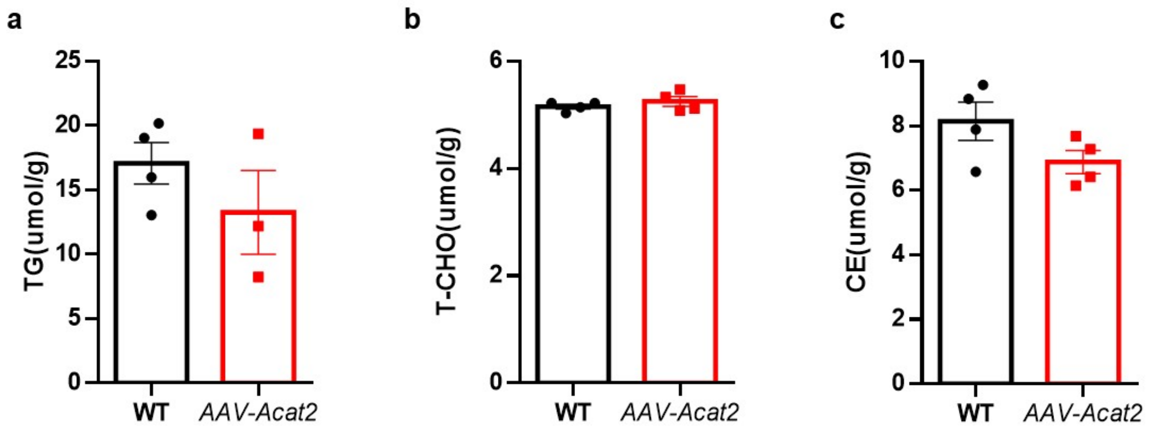

ESM Fig. 4 (a-c) the relative amount of TG (a), cholesterol (b) and CE (c) from liver of control and *AAV9-Acat2* injected mice.

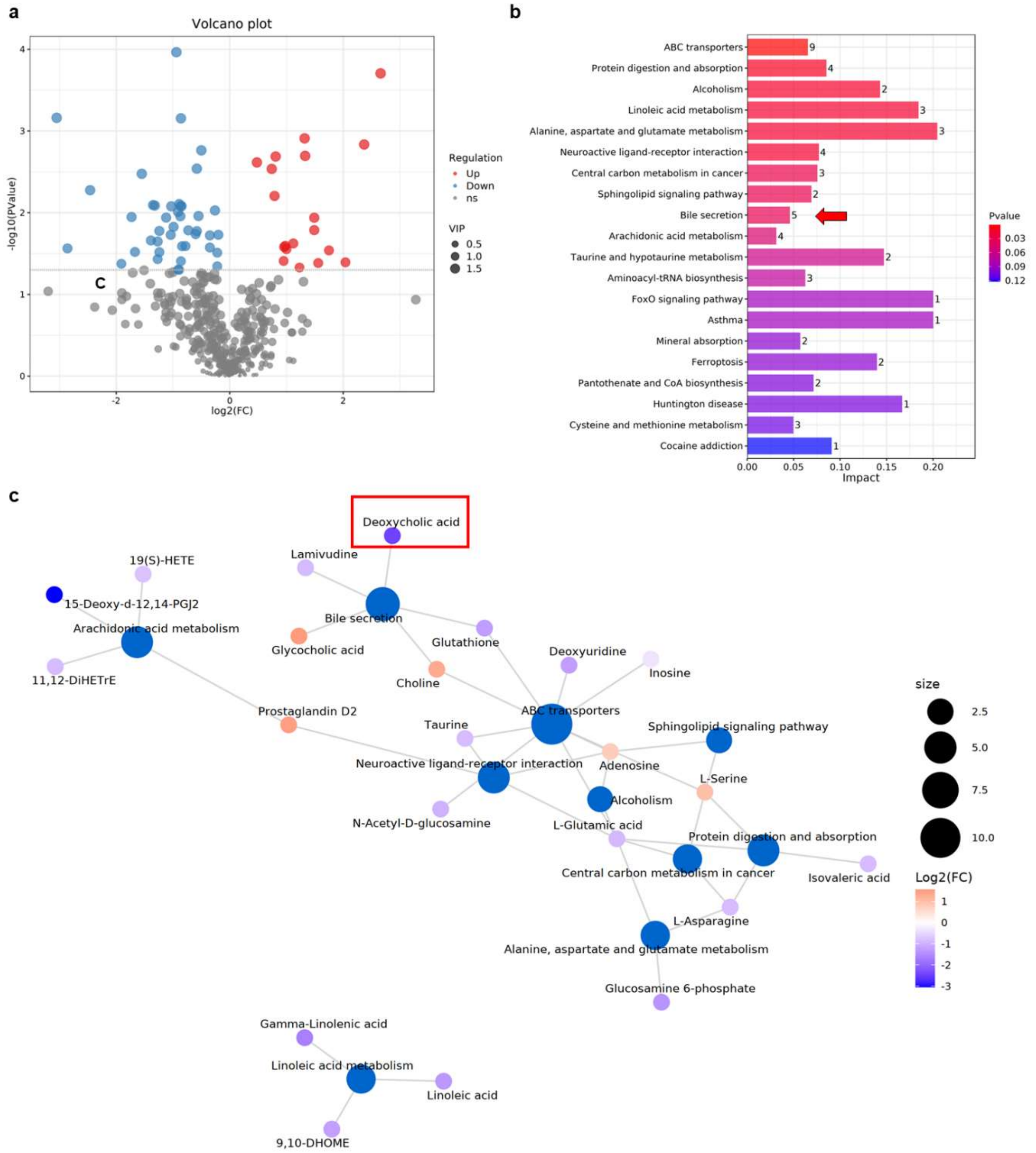

ESM Fig. 5 (a) Volcano plot showing differential metabolites from liver of *Acat2* overexpressed and control mice. Red dots represent up-regulated metabolites in *AAV9-Acat2* injected mice and blue dots represent the down-regulated ones. (b, c) KEGG annotation to identity the key metabolic pathways changed in *Acat2* overexpressed liver, including bile secretion.

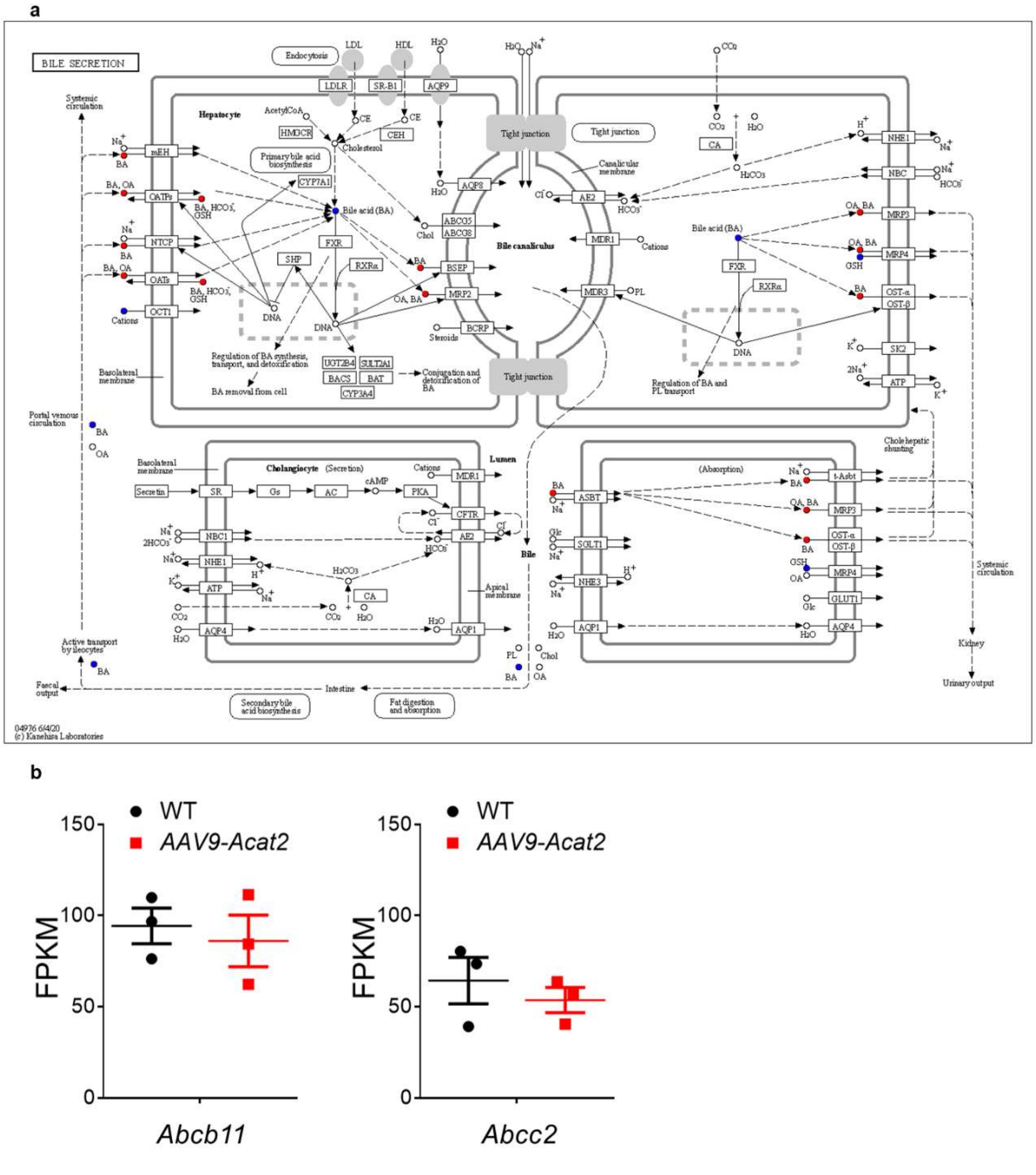

ESM Fig. 6 (a) Metabolic changes in bile secretion. Red dots represent up-regulated metabolites in *AAV9-Acat2* injected mice and blue dots represent the down-regulated ones. (b) FPKM showing the relative gene expression levels of two bile acid transporter, *Abcb11* and *Abcc2*.

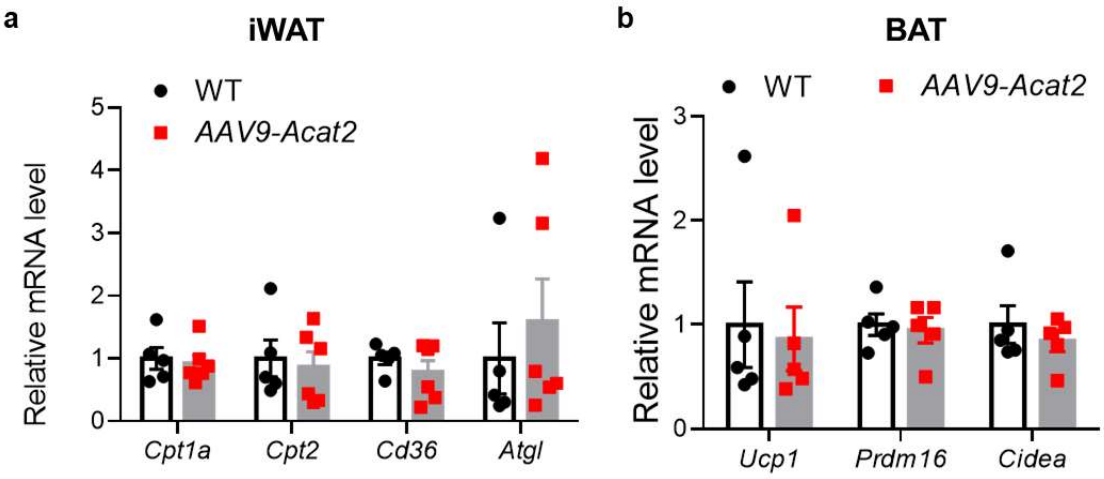

ESM Fig. 7 (a, b) Relative levels of genes involved in FA transport, Lipolysis, β-oxidation and thermogenesis of iWAT (a) and BAT (b) from control and *AAV9-Acat2* injected mice. Data represent mean±s.e.m.

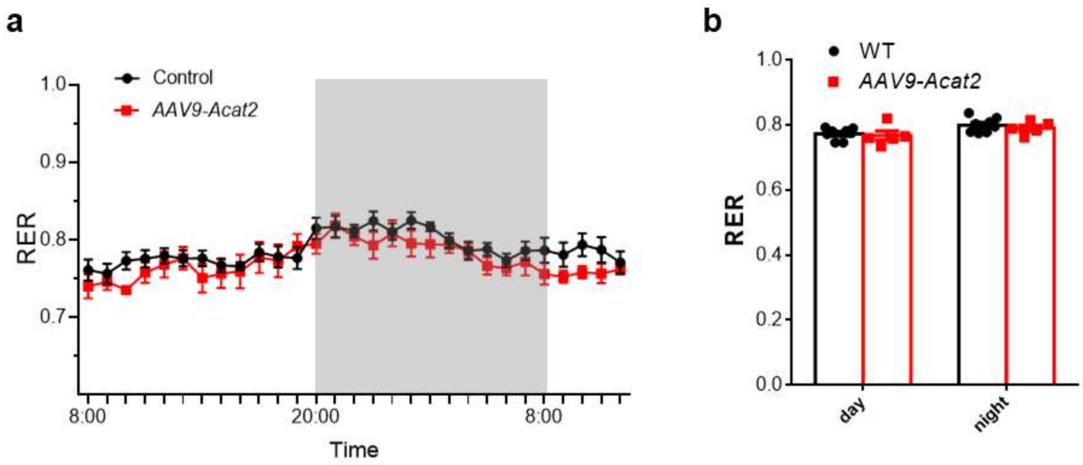

ESM Fig. 8 (a, b) Respiratory exchange rate (a) and average day and night RER (b) are calculated of WT mice control and *AAV9-Acat2* injected mice after high fat diet treatment.

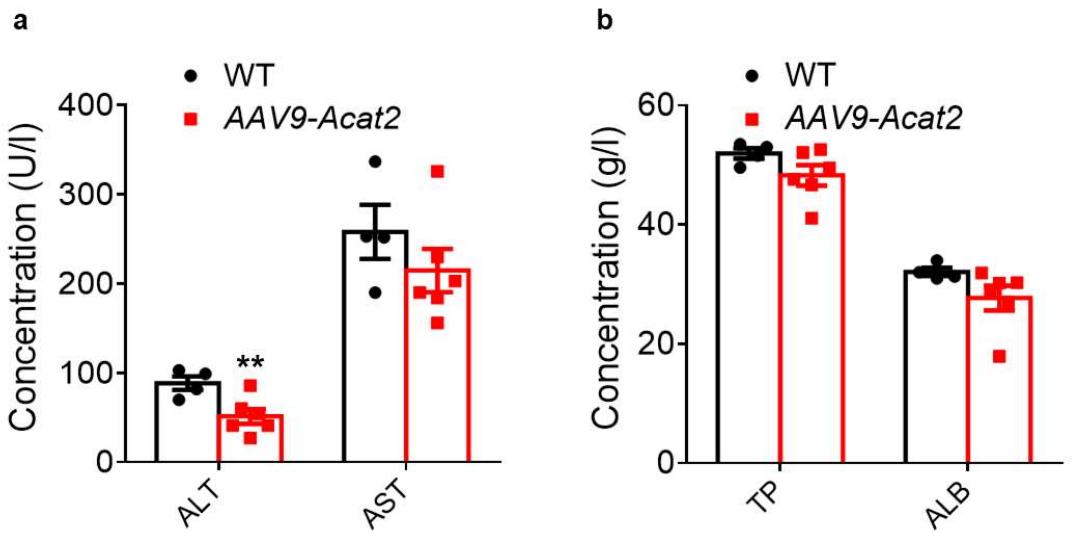

ESM Fig. 9 (a, b) Concentrations of ALT and AST (a), TP and ALB (b) from the serum of control and *AAV9-Acat2* injected mice after 10-week of high fat diet treatment. N=4 and 6 male mice control and *AAV9-Acat2* groups, respectively. Data represent mean±s.e.m. (t-test: ** P<0.01).
